# Gastric cancer secreted miR-214-3p inhibits the anti-angiogenesis effect of apatinib by suppressing ferroptosis in vascular endothelial cells

**DOI:** 10.32604/or.2023.046676

**Published:** 2024-02-06

**Authors:** WEIXUE WANG, TONGTONG WANG, YAN ZHANG, TING DENG, HAIYANG ZHANG, YI BA

**Affiliations:** Department of GI Medical Oncology, Tianjin Medical University Cancer Institute and Hospital, National Clinical Research Center for Cancer, Tianjin’s Clinical Research Center for Cancer, Tianjin Key Laboratory of Digestive Cancer, Key Laboratory of Cancer Prevention and Therapy, Tianjin, 300060, China

**Keywords:** Cellular ferroptosis, Exosome, Tyrosine kinase inhibitor, Gastrointestinal tumors, miRNA

## Abstract

Different from necrosis, apoptosis, autophagy and other forms of cell death, ferroptosis is a mechanism that catalyzes lipid peroxidation of polyunsaturated fatty acids under the action of iron divalent or lipoxygenase, leading to cell death. Apatinib is currently used in the third-line standard treatment of advanced gastric cancer, targeting the anti-angiogenesis pathway. However, Apatinib-mediated ferroptosis in vascular endothelial cells has not been reported yet. Tumor-secreted exosomes can be taken up into target cells to regulate tumor development, but the mechanism related to vascular endothelial cell ferroptosis has not yet been discovered. Here, we show that exosomes secreted by gastric cancer cells carry miR-214-3p into vascular endothelial cells and directly target zinc finger protein A20 to negatively regulate ACSL4, a key enzyme of lipid peroxidation during ferroptosis, thereby inhibiting ferroptosis in vascular endothelial cells and reducing the efficiency of Apatinib. In conclusion, inhibition of miR-214-3p can increase the sensitivity of vascular endothelial cells to Apatinib, thereby promoting the antiangiogenic effect of Apatinib, suggesting a potential combination therapy for advanced gastric cancer.

## Introduction

Ferroptosis is caused by the redox imbalance between the products of oxidants and antioxidants whose sensitivity is closely related to numerous biological processes, mainly including polyunsaturated fatty acid metabolism and production and degradation pathways (Lysosomal pathway and the ubiquitin-proteasome system) of essential proteins [1–3]. It is driven by the abnormal expression and activity changes of a variety of redox-active enzymes, which produce free radicals and lipid oxidation products or detoxify them [4,5]. Several metabolic pathways, such as thiol metabolism, fatty acid metabolism, and iron metabolism, directly affect the sensitivity of cells to lipid peroxidation and ferroptosis [2]. Recently, multiple cell-intrinsic proteins have been reported to regulate the occurrence and process of ferroptosis. Acyl-CoA synthetase long-chain family member 4 (ACSL4), lysophosphatidylcholine acyltransferase 3 (LPCAT3) and arachidonic acid lipoxygenase (ALOX) are involved in PUFA metabolism [6,7]. Among them, ACSL4 preferentially activates long-chain polyunsaturated fatty acids used for phospholipid synthesis, and it is responsible for shaping the cellular lipidome [8].

Apatinib is a selective vascular endothelial growth factor receptor-2 (VEGFR-2) tyrosine kinase inhibitor [9]. In 2016, Li et al. reported the results of a phase III trial that evaluated Apatinib treatment in patients who was suffering advanced gastric cancer and made a landmark step forward in gastric cancer targeted therapy [10]. A series of combined clinical trials were also followed, such as the application of SHR-1210 (anti-PD-1 antibody) as combination therapy in patients with gastric or esophagogastric junction cancer (GC/EGJC) [11]. As third-line therapy, Apatinib significantly prolonged median overall survival (OS) and progression-free survival (PFS), but the problem of drug resistance also appeared [12,13]. A similar drug, sorafenib, has been shown to have a functional synergistic antiangiogenic effect as a ferroptosis inducer in advanced hepatocellular carcinoma. In addition to targeting VEGFR-2 [14], Apatinib can also induce apoptosis [15,16], autophagy [16], stem cell properties [17], ferroptosis by lipid peroxidation [18] in gastric cancer cells to play a therapeutic role.

Exosomes are a subtype of extracellular vehicles (EVs) with a typical size approximately from 40 to 160 nm in diameter [19]. Exosomes are secreted by multiple cell types, in which tumor cells have been found to produce significantly, playing different roles in regulating tumor growth and progression, invasion and metastasis, neovascularization and immune escape [20,21], called Tumor-derived exosomes (TDEs). The mechanisms of TDEs affecting drug resistance in chemotherapy, targeted therapy and immunotherapy in advanced gastric cancer are occasionally reported [22–27]. Several studies have demonstrated that exosomes play an important role in inducing ferroptosis or driving ferroptosis resistance [28,29]. For example, it is associated with immune checkpoint blockade drug resistance of tumor cells [30,31], chemotherapeutic drug resistance [31,32], or the application of engineered extracellular vesicles to inhibit tumor growth by reprogramming the tumor microenvironment and regulating ferroptosis [33,34], or derived from vascular endothelial cells The exosomes are also strongly linked to ferroptosis [35–37]. This may open up new therapeutic opportunities for modulating ferroptosis in human pathology.

In this study, we found that ferroptosis of vascular endothelial cells is significantly inhibited in advanced gastric cancer, which contributes to tumor angiogenesis and reduces the sensitivity to Apatinib. And A20 is a ubiquitin editing enzyme that regulates the degradation of ACSL4, of which mechanism can be observed and is closely related to ferroptosis of vascular endothelial cells. In addition, exosomal miR-214-3p secreted from GC cells play a leading role in regulating the expression of A20 in vascular endothelial cells. Therefore, this study identified a new pathway to regulate the ferroptosis of vascular endothelial cells mediated by GC cell exosomes and provided a new idea to enhance the sensitivity of third-line Apatinib targeted therapy for advanced gastric cancer.

## Materials and Methods

### Human tissues

All the tumor tissue samples and plasma samples of GC patients were obtained from Tianjin Medical University Cancer Institute and Hospital. Our study was approved by the Ethics Committee of Tianjin Medical University Cancer Institute and Hospital. Informed consent was obtained from the patient to publish this study. All the study methodologies conformed to the standards set by the Declaration of Helsinki. And the key inclusion criteria were: age of ≥18 years; histologically confirmed metastatic or recurrent gastric or gastroesophageal junction (GEJ) adenocarcinoma; previous first-line use of platinum combined with fluorouracil or disease progression within 6 months after withdrawal of adjuvant chemotherapy with platinum and fluorouracil-based regimen, or failure of second-line irinotecan or paclitaxel.

### Cell culture

Human Umbilical Vein Endothelial Cell (HUVEC) were cultured in DMEM medium (Gibco, Grand Island, NY, USA). Human gastric cancer cell lines, MKN45, HGC27 cells were bought from cell bank of Chinese Academy of Sciences (Shanghai, China) and were cultured in 1640 (Gibco). All cell lines were tested for mycoplasma contamination and were supplemented with 10% fetal bovine serum (FBS) (Gibco) and 1% penicillin/streptomycin (Solarbio) in a humidified incubator at 37°C with 5% CO_2_.

### Animals

Female nude mice (BALB/c-nu, 4 weeks) purchased from GemPharmatech Co., Ltd. (Jiangsu, China) were fed in a special pathogen-free animal facilities with access to eat and drink ad libitum. All the experimental process were performed as protocols approved by Tianjin Medical University Cancer Institute and Hospital.

### Vascular ring formation of HUVEC cells

Melt the Matrigel in advance and add 100 μl to each well of the 24-well plate, placing at 37°C for 30 min to solidify. HUVEC cells were first co-cultured with 25 μmol Apatinib. Then the cells were resuspended in DMEM and seeded at a concentration of 10^5^ cells per well. The formation of capillary-like structures was examined under a light microscope after 6 h.

### Nanoparticle tracking analysis (NTA)

The density and size of exosomes were tracked by the Nanosight NS300 system (NanoSight Technology, Malvern, UK). Exosomes were re-suspended in PBS at a concentration of 5 μg/mL and then further diluted 100- to 500-fold to achieve between 20 and 100 objects per frame. Samples were manually injected into the sample chamber at ambient temperature. Each sample was configured with a 488 nm laser and a high-sensitivity sCMOS camera. At least 200 completed tracks were analyzed per video. All of the data were analyzed via the NTA analytical software (version 2.3; Thery and Witwer, 2018).

### Induction and inhibition of ferroptosis

To induce ferroptosis, HUVEC were seeded in 6-well plates with suitable density. Erastin was used as the ferroptosis inducing compounds and Ferrostatin-1, Lipostatin-1 were used as the ferroptosis inhibitor, which were purchased from Sigma-Aldrich (St. Louis, MO, USA).

### PKH26 staining assay

The PKH26 Red Fluorescent Cell Linker Kit (Sigma) was used for exosome staining. 50 mg exosomes resuspended in 100 μL diluent C which was mixed with 100 μL PKH26 dye solution and incubated for 10 min before stopped by adding 200 μL serum. Labeled exosomes were washed twice using PBS and co-incubated with HUVEC for 4–8 h.

### Western blotting

Protein samples were subjected to SDS-PAGE. The expression of GPX4, A20, ACSL4 were assessed by western blotting analysis and samples were normalized to β-actin. Protein extraction was blocked with 5% skimmed milk powder at room temperature for 1 h and incubated at 4°C overnight with anti-GPX4 (1:5000, Santa Cruz), anti-A20 (1:1000, Santa Cruz), anti-ACSL4 (1:1000, Santa Cruz), anti-CD81 (1:1000, Santa Cruz), anti-TSG101 (1:1000, Santa Cruz), anti-CD9 (1: 2000, CST), anti-ubiquitin (1:1000, Abcam), and anti-β-actin (1:5000, Santa Cruz) antibodies, respectively.

### RT-qPCR

Total RNA was extracted with TRIzol reagent (Invitrogen) from cells, exosomes and tissues, and then utilized for reverse transcription PCR (Eppendorf AG 22331 Hamburg, Germany) to synthesize cDNA used for real-time qPCR (Bio-Rad CFX96, Hercules, CA, USA). Quantification of miR-214-3p was performed and normalized to the internal control U6 small nuclear RNA and mRNA levels were normalized to GAPDH.

The relative amount of gene normalized to control was calculated with the equation 2^−ΔCT^, in which ΔCT  =  CT gene-CT control.

A20 primers

Forward: 5′-CTGCTGGCTGCCTGTCTCAAG-3′

Reverse: 5′-GTTCTGGAACCTGGACGCTGTG-3′

ACLS4 primers

Forward: 5′-AGAATACCTGGACTGGGACCGAAG-3′

Reverse: 5′-TGCTGGACTGGTCAGAGAGTGTAA-3′

### Lipid ROS levels

Following different treatments, cells were stained with 10 μM C11-BODIPY581/591 probe (Invitrogen) for 30 min and was used to detect the level of lipid ROS by flow cytometry according to the manufacturer’s protocol. Analysis of C11-BODIPY581/591 fluorescence was performed by a BD Accuri C6 flow cytometer.

### CCK-8 assay

Cells were inoculated in 96-well plates. After treatment with the different conditions, cell viability was measured using the Cell Counting Kit-8 (CCK-8, Biosharp, China) assay. 10 μl CCK-8 reagent was added to each well and the cells were incubated further for 2–4 h at 37°C. The optical density value was measured at 450 nm. The following formula was used to calculate the cell inhibiting rate: Cell inhibiting rate (%) = [(Ac − Ae)/(Ac − Ab)] × 100% (Ac = the absorbance of the control well, Ae = the absorbance of the experimental well, Ab = the absorbance of the blank well).

### ACSL4 mRNA half-life determination

To determine ACSL4 mRNA half-life, HUVEC cells were treated with 150 μM of Apatinib (or DMSO) or infected with A20 siRNA for 24 h. Actinomycin D (5 μg/ml) (Sigma Aldrich, a4262) was added to cells at different intervals (0, 2, 4, 8, and 24 h). At the end of incubation, total mRNA was examined.

### ACSL4 protein half-life determination

To determine ACSL4 protein half-life, HUVEC cells were treated with 150 μM of Apatinib (or DMSO) for 24 h or infected with A20 siRNA for 48 h. Cycloheximide (CHX) (5 μg/ml) (Sigma Aldrich, c7698) was added to cells at different intervals (0, 2, 4, 8, and 24 h). At the end of incubation, total protein was harvested followed by Western blotting analysis.

### Ubiquitination assay

A20 siRNA was transfected into HUVEC cells. At 48 h after transfection, cells were treated with 20 μM of the proteasomal inhibitor MG132 for 6 h before cell lysis. ACSL4 protein was separated by co-immunoprecipitation method and subjected to SDS electrophoresis and western blotting analysis, detected with Ub antibody (Abcam, ab134953).

### GSH analysis

GSH was detected with the GSH detection kit. Briefly, HUVEC cells were seeded into 6-well cell culture plates with 2 * 10^5^ cells per well and processed differently. Cells were harvested and washed twice with PBS. Mix 0.5 ml of the sample with 2 ml of reagent I, centrifuge at 3500–4000 rpm for 10 min. 1 ml of the supernatant fluid was detected at the wavelength of 420 nm.

### BODIPY-493/503 staining assay

HUVEC cells were pretreated with Apatinib (Jiangsu Hengrui, China, 50 μM) and Arachidonic acid (AA, 50 μM) for 6/24 h. Then cells were stained with BODIPY-493/503(1 µg/mL) for 30 min and DAPI (1:1000 diluted by 1 * PBS) for 10 min and imaged by confocal microscopy (Zeiss, Jena, Germany).

### IHC staining assay

Animal tumor tissues were sectioned and stained with a 1:1000 dilution of anti-ACSL4 antibody (Abcam), 1:1000 dilution of anti-A20 antibody (Abcam), 1:500 dilution of anti-CD34 antibody (Abcam). Five regions were selected randomly for each specimen.

### Statistical analyses

All experiments were repeated at least three times in parallel. *p* value < 0.05 was considered statistically significant by using the student’s *t*-test: *p < 0.05, **p < 0.01, ***p < 0.001 and ****p < 0.0001.

## Results

### Apatinib induces ferroptosis by increasing ACSL4 in vascular endothelial cells

**Figure 1:**
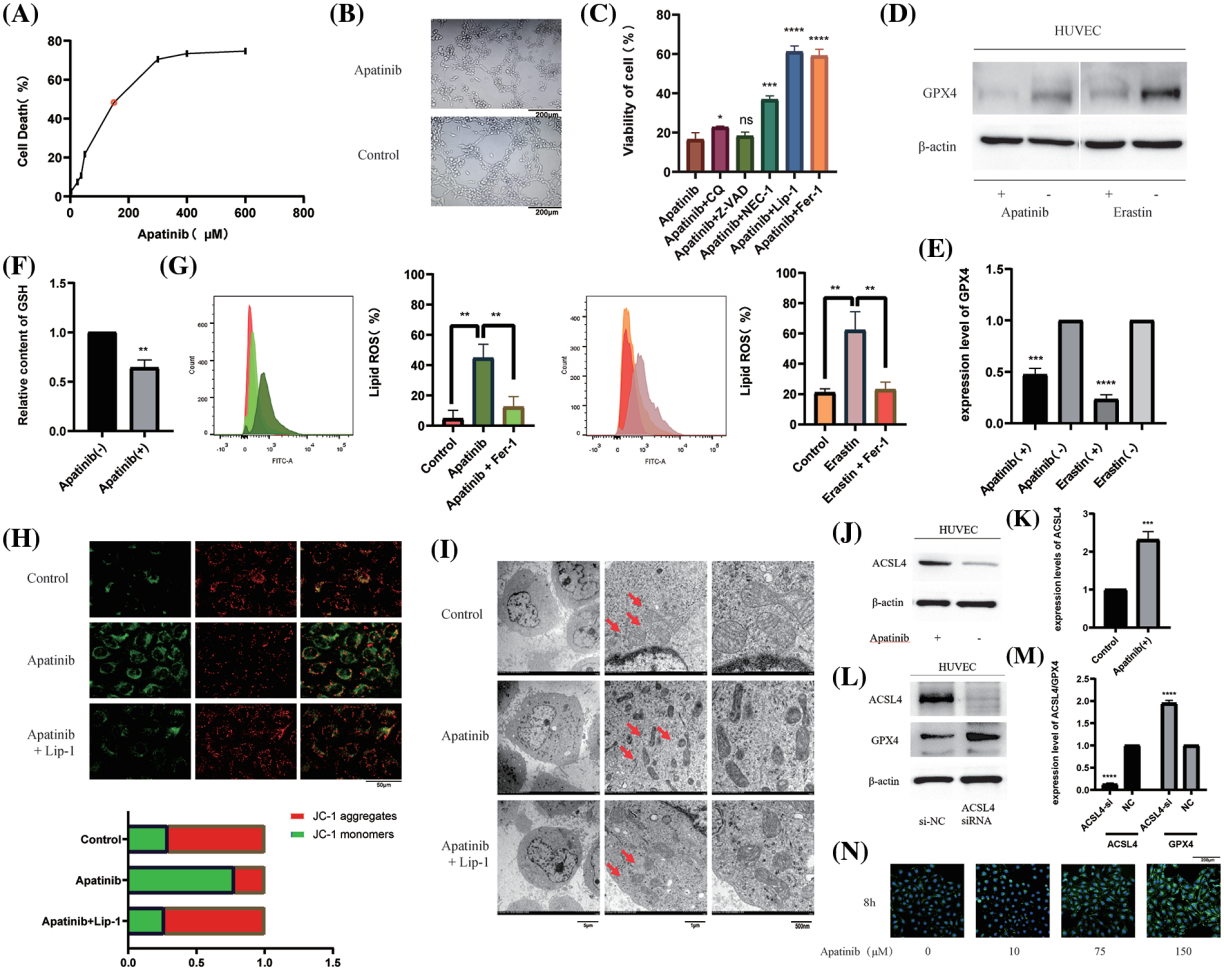
Apatinib induces ferroptosis by increasing ACSL4 in vascular endothelial cells. (A) IC50 of Apatinib was determined by CCK-8 assay. (B) Apatinib affects the vascular looping function of HUVECs. (C) Cell death by Apatinib can be reversed by the ferroptosis inhibitors Lip-1 and Fer-1 by CCK-8. (D, E) Apatinib and Erastin could affect GPX4 expression in HUVEC, respectively. (F) Apatinib reduces intracellular GSH content. (G) Apatinib and Erastin, respectively, can increase the accumulation of intracellular lipid ROS and are reversed to some extent by Fer-1. (H) Apatinib reduces intracellular mitochondrial membrane potential and is reversed to some extent by Fer-1. (I) Apatinib shrinks intracellular mitochondria under electron transmission microscopy and is reversed to some extent by Lip-1. (J, K) Apatinib increases ACSL4 expression in HUVECs. (L, M) Increased intracellular GPX4 expression after transfection with ACSL4 siRNA. (N) Treatment of HUVECs with different concentrations of Apatinib for 8 h altered the rate of cellular fatty acid uptake (*p < 0.05; **p < 0.01; ***p < 0.001; ****p < 0.0001).

In order to verify the effect of Apatinib on vascular endothelial cells, we first treated HUVEC with different concentrations of Apatinib to obtain a suitable treatment concentration of 150 μM (Fig. 1A). At this concentration, Apatinib affected vascular ring formation *in vitro* (Fig. 1B). To explore the type of cell death caused by Apatinib, we applied various types of cell death inhibitors and found that the cell death caused by Apatinib can be reversed to a certain extent by the ferroptosis inhibitors Fer-1 and Lip-1 (Fig. 1C). Except for that, Nec-1 can also reverse cell death caused by Apatinib, indicating that there happens necrosis in HUVEC as well. The specific mechanism is not yet clear, and we will continue to explore relevant mechanisms in the follow-up research. In this study, we mainly investigated the ferroptosis of HUVEC caused by Apatinib. In order to further verify that Apatinib does indeed cause ferroptosis in HUVEC, we found through a series of experiments that Apatinib can play a similar role to Erastin, as shown in the western blot to verify that it can affect the expression of GPX4 (Figs. 1D and 1E), though we did not find out the specific mechanisms between Apatinib or Erastin and GPX4, which is an important enzyme in the process of ferroptosis. Apatinib can also reduce GSH levels (Fig. 1F) and increase intracellular lipid ROS (Fig. 1G). Apatinib decreased intracellular mitochondrial membrane potential as observed by JC-1 fluorescence staining, which existed as a green fluorescent monomer under the microscope (Fig. 1H). Apatinib-treated HUVEC exhibited morphological changes typical of ferroptosis as observed by transmission electron microscopy (Fig. 1I, Suppl. Figs. 1A and 1B).

Studies have shown that Apatinib can regulate the expression of ACSL4 in colorectal cancer cells [38]. To verify the pathway by which Apatinib regulates ferroptosis in HUVEC, we treated vascular endothelial cells with 150 μM Apatinib for 24 h. By western blot, we found that it increased the expression of ACSL4 (Figs. 1J and 1K). Surprisingly, we found that ACSL4 siRNA could affect the expression of GPX4 after transfection into cells (Figs. 1L and 1M) and the mechanisms are not very clear. Based on the function of ACSL4 to catalyze lipid peroxidation in the ferroptosis mechanism, we verified the effect on intracellular fatty acid uptake with increasing Apatinib concentration. Through BODIPY-493/503 staining assay we verified that the accumulation of intracellular lipid droplets increased with increasing Apatinib treatment concentrations (Fig. 1N). Our results confirm that Apatinib can induce ferroptosis in vascular endothelial cells and affect fatty acid uptake by regulating ACSL4.

### Identifying A20 as upstream of ACSL4 and regulating its degradation through deubiquitination

**Figure 2:**
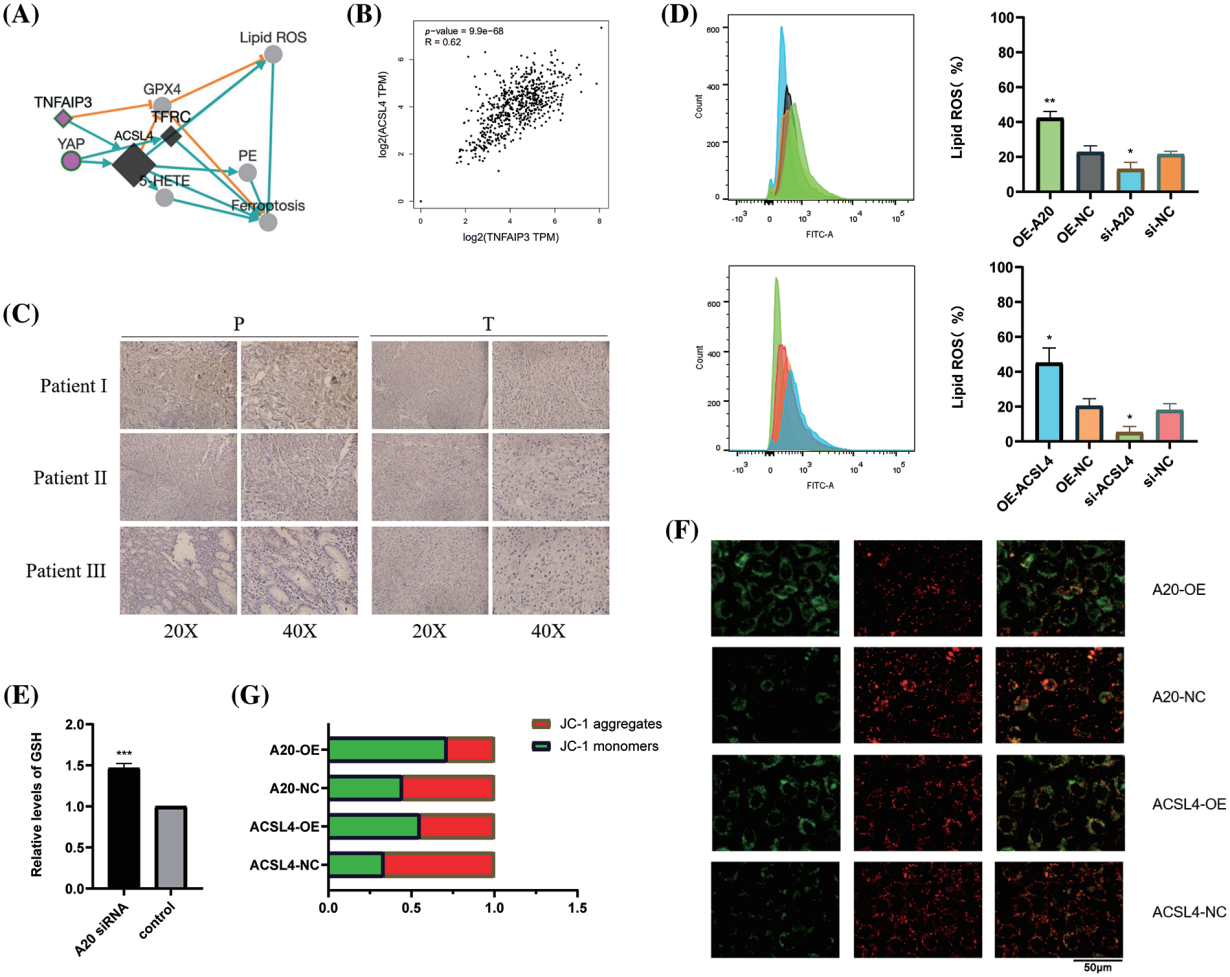
Identifying A20 as upstream of ACSL4. (A) ACSL4 association network predicted by FerrDb database. (B) Correlation of TNFAIP3 with ACSL4 was determined by GEPIA database. (C) Immunohistochemical staining of TNFAIP3 in cancer tissues and adjacent normal tissues. (D) The effect of A20 on intracellular lipid ROS accumulation was detected by flow cytometry. (E) Increased intracellular GSH content after transfection of A20 siRNA. (F, G) Effects of A20 and ACSL4 on intracellular mitochondrial membrane potential (*p < 0.05; **p < 0.01; ***p < 0.001; ****p < 0.0001).

**Figure 3:**
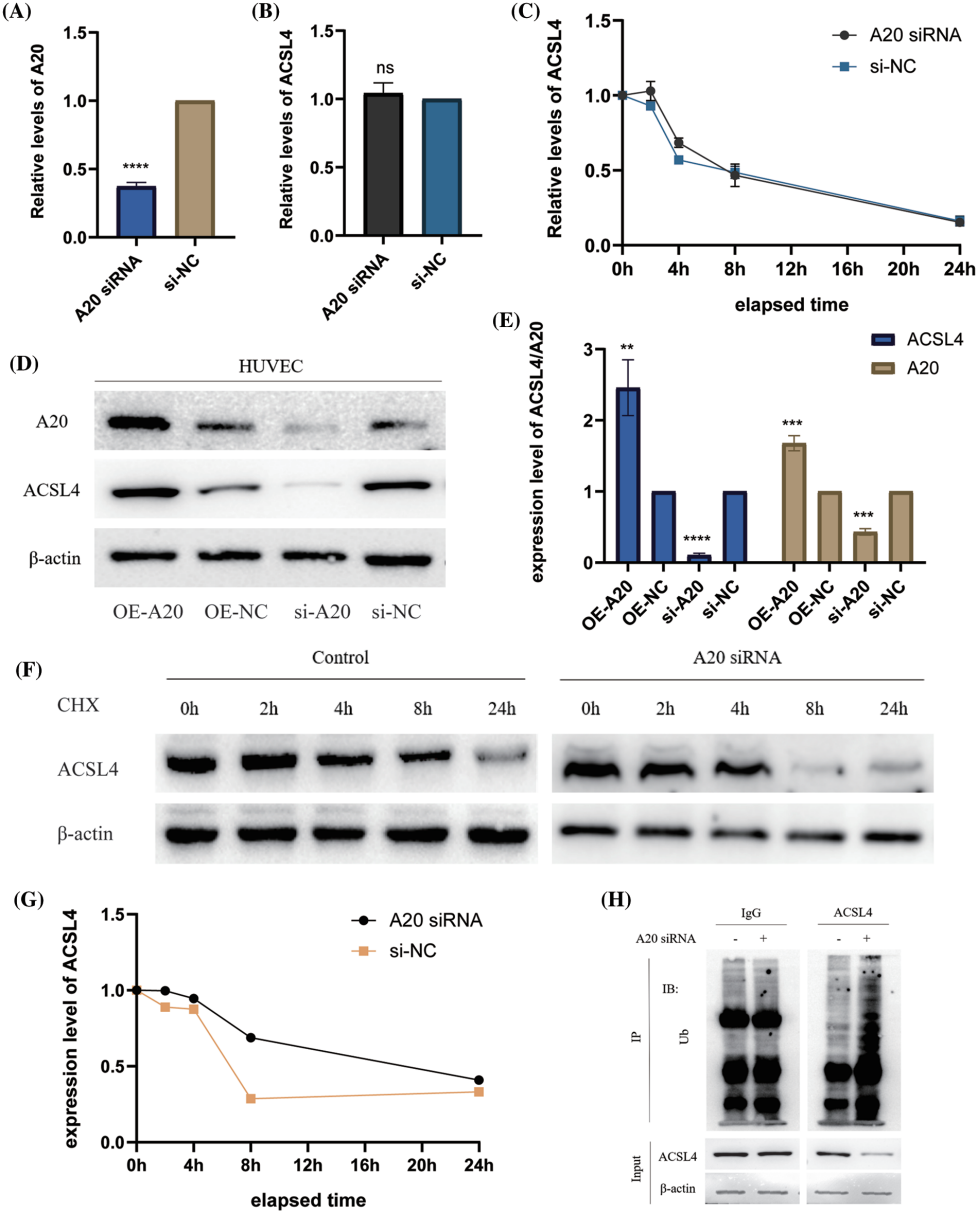
A20 regulating ACSL4 degradation through deubiquitination. (A) PCR verification of the efficiency after transfection of A20 siRNA. (B) PCR to verify the effect on ACSL4 mRNA after transfection of A20 siRNA. (C) PCR validation of the effect on ACSL4 mRNA half-life within 24 h after transfection of A20 siRNA. (D, E) Western blotting to verify the effect on ACSL4 protein after transfection of A20 siRNA. (F, G) Western blotting to verify the effect on ACSL4 protein half-life within 24 days after transfection of A20 siRNA. (H) IP assay to verify the ubiquitination effect of A20 siRNA on ACSL4 (*p < 0.05; **p < 0.01; ***p < 0.001; ****p < 0.0001).

To identify the upstream regulators of ACSL4, we identified the gene interaction network associated with ACSL4 through the FerrDb database and predicted TNFAIP3 (A20) as a regulator of ACSL4 (Fig. 2A). Through the GEPIA database we determined the co-expression of ACSL4 and A20 in gastric adenocarcinoma (Fig. 2B). We collected sections of cancer tissue and paracancerous tissue from eight patients with advanced gastric cancer and performed immunohistochemical experiments to verify that the expression of A20 in cancer tissue was significantly lower than that in paracancerous tissue (Fig. 2C, Suppl. Fig. 2A), and the specific mechanism is still unclear. And because of the quality of the sections, the difference in the expression of A20 in vascular endothelial cells cannot be explained yet. Here we just make a guess. To further verify the relationship between A20/ACSL4 and HUVEC ferroptosis, we verified through a series of functional assays that overexpression of A20/ACSL4 can reduce the accumulation of intracellular lipid ROS (Fig. 2D), increase GSH levels (Fig. 2E) and reduce intracellular Mitochondrial membrane potential (Figs. 2F and 2G). Next, we transfected A20 siRNA into cells and verified its transfection efficiency by PCR (Fig. 3A). In order to further explore the mechanism of A20 regulating ACSL4, we verified from the mRNA level and the protein level. We found that transfection of A20siRNA had little effect on ACSL4 mRNA by PCR (Fig. 3B). By adding actinomycin D to inhibit the synthesis of intracellular RNA, we found that transfection of A20 siRNA also had little effect on the half-life of ACSL4 mRNA (Fig. 3C). It was demonstrated that A20 may have no effect on ACSL4 at the transcriptional level. At the protein level, we found that transfection of A20 overexpression plasmid or A20 siRNA into HUVEC cells could affect ACSL4 protein expression (Fig. 3D and 3E). By adding cycloheximide to inhibit intracellular protein synthesis, we found that transfection of A20 siRNA could prolong the half-life of ACSL4 protein (Figs. 3F and 3G). demonstrated that A20 may affect ACSL4 at the post-translational level. Through ubiquitination assay, we found that transfection of A20 siRNA could increase the ubiquitinated ACSL4 (Fig. 3H), indicating that the presence of A20 can protect ACSL4 from being ubiquitinated and degraded.

### Construction of differentially expressed miRNA profiles in advanced gastric cancer and identification of the upstream of A20

**Figure 4:**
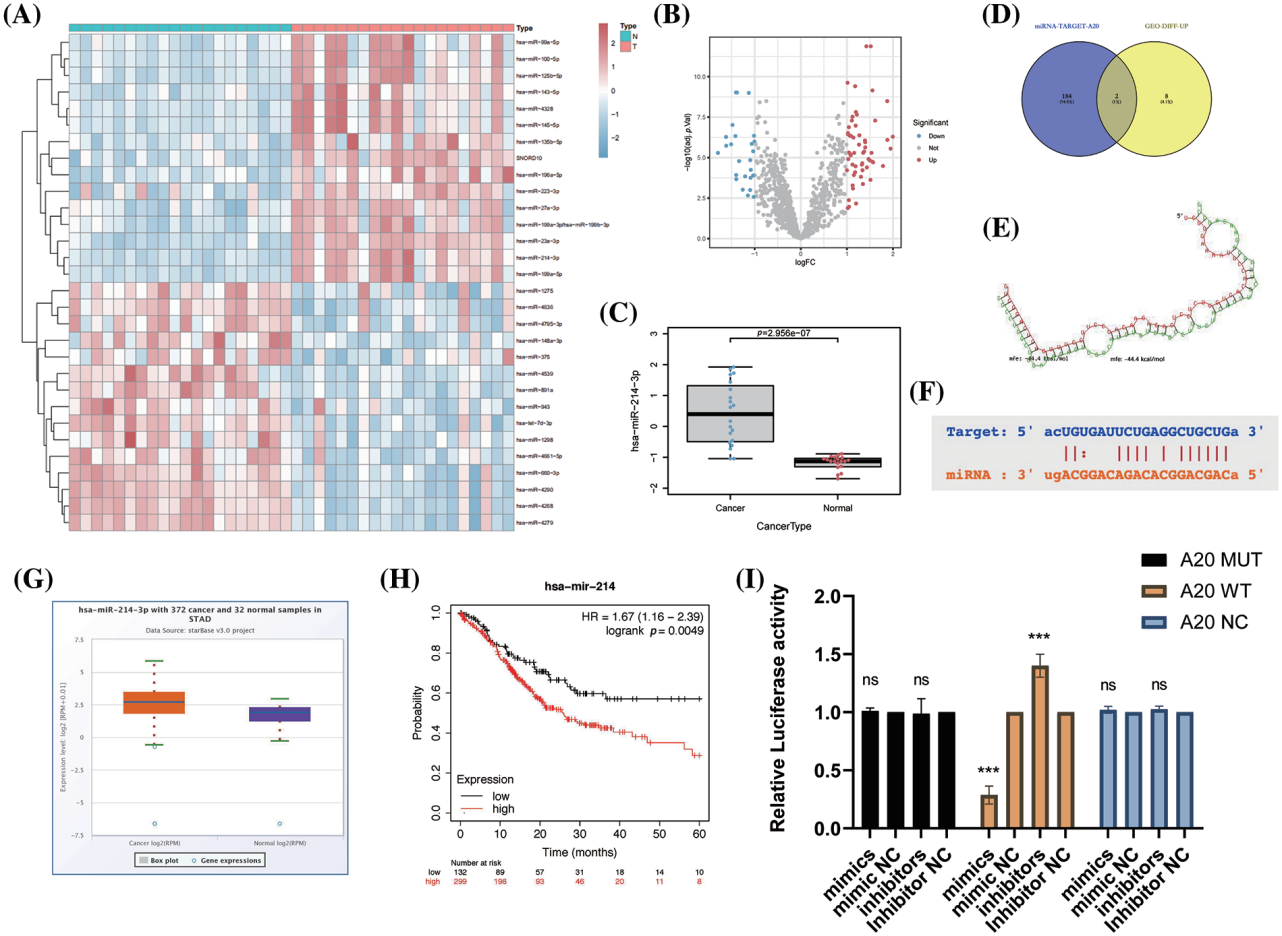
Construction of differentially expressed miRNA profiles in advanced gastric cancer and identification of the upstream of A20. (A–C) Differentially expressed miRNA profiles in gastric cancer tissues and adjacent healthy gastric mucosa in the GEO database. (D) Venn diagram drawn by Venny2.1.0. (E) The direct binding region of miR-214-3p constructed by RNA Hybrid to A20. mfe: −44.4 kcal/mol. (F) PicTar validates the region where miR-214-3p binds directly to A20. (G) Differential expression of miR-214-3p in gastric adenocarcinoma and normal tissues. (H) Correlation of miR-214-3p with prognosis in gastric adenocarcinoma. (I) Dual-luciferase reporter gene to verify the direct binding of miR-214-3p to A20 (*p < 0.05; **p < 0.01; ***p < 0.001; ****p < 0.0001).

We found the miRNA database (GSE93415) in advanced gastric cancer in the GEO database and performed data exploration, constructed the differentially expressed miRNA profiles in gastric cancer tissue and adjacent healthy gastric mucosa, and drew heat maps, volcano plots and histograms to visualize the data (Figs. 4A–4C, Suppl. Fig. 3A). After this, we crossed the top 10 miRNAs whose expression was up-regulated with all miRNAs predicted to be directly related to A20 in the TargetScan database (Fig. 4D). We found two miRNAs, miR-23a-5p and miR-214-3p, that share two roles. Since our research team has previously studied the relevant role of miR-214-3p in oxaliplatin resistance of colorectal cancer, we are more interested in other roles of miR-214-3p, so we chose it as the target of follow-up research. The role of miR-23a-5p in regulating A20 and subsequent processes will be studied in detail in the future, and will not be focused here. The ability of miR-214-3p to directly bind to A20 was verified by RNA Hybrid and PicTar tools (Figs. 4E and 4F). The expression of miR-214-3p in cancer tissues of patients with advanced gastric cancer was significantly higher than that in adjacent tissues (Fig. 4G) and was significantly correlated with prognosis (Fig. 4H). Finally, we verified the direct binding of miR-214-3p to A20 by dual-luciferase reporter experiments (Fig. 4I).

### miR-214-3p is derived from gastric cancer cell exosomes and regulates ferroptosis in HUVEC

**Figure 5:**
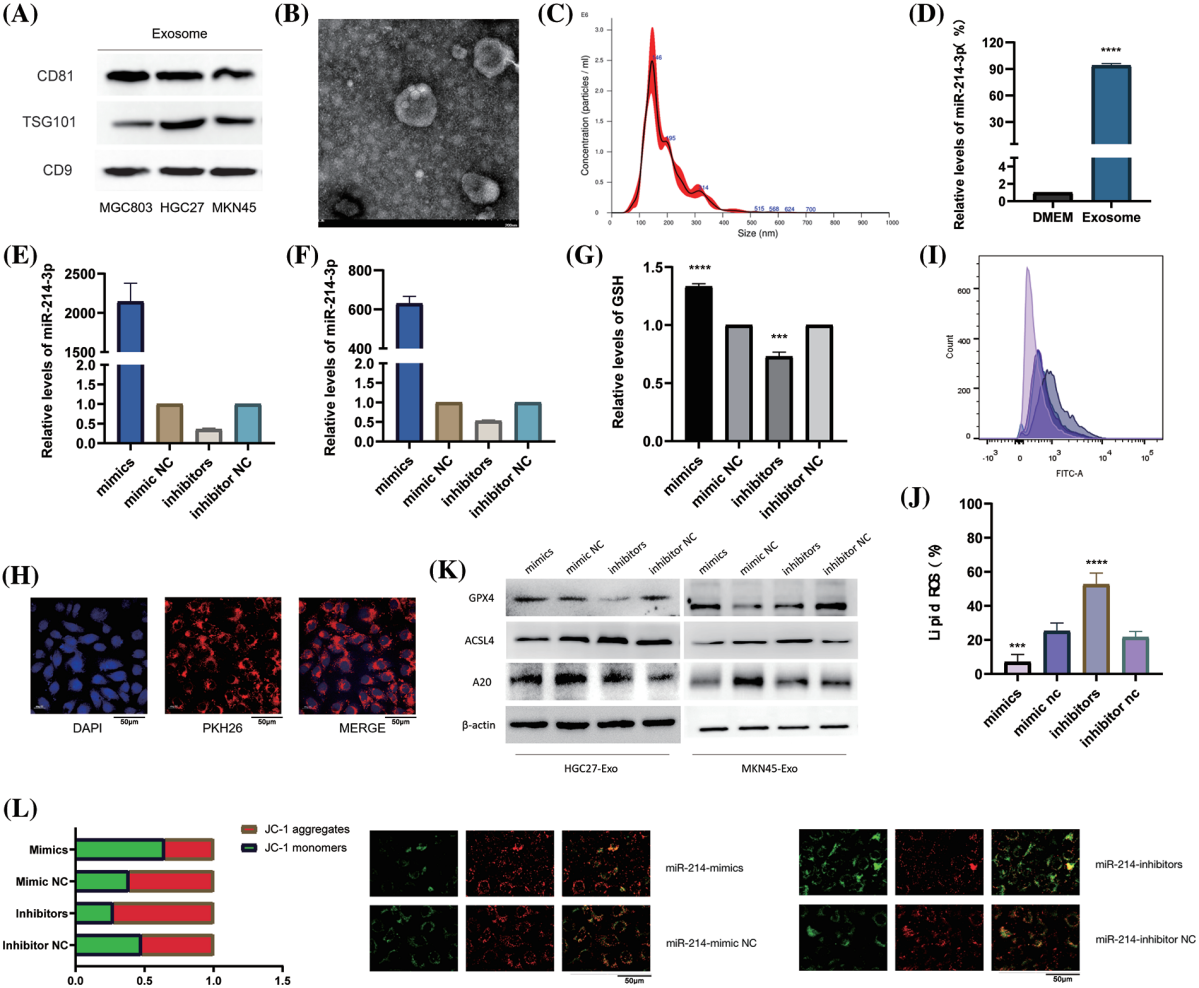
miR-214-3p is derived from gastric cancer cell exosomes and regulates ferroptosis in HUVEC. (A) The markers of exosomes secreted by HGC27 and MKN45 gastric cancer cells were verified by western blotting. (B) Exosomes under electron microscope. (C) The density and size of exosomes were tracked by the Nanosight NS300 system. (D) PCR verification of miR-214-3p content in exosome-removed culture medium and exosomes. (E–G) Transfection efficiency after transient transfection of miR-214-3p mimics and inhibitors and their control groups into HGC27 and MKN45 cells, respectively, and extraction of exosomes and co-culture with HUVECs for 24 h were verified by PCR. (H) Validation of exosomes uptake by HUVECs by PKH26 staining. (I) Gastric cancer exosomes extracted after transfection can affect the GSH content of HUVECs. (J) Gastric cancer exosomes extracted after transfection were able to affect lipid ROS accumulation in HUVECs. (K) It was verified by western blot that the exosomes extracted from the two cells could affect the expression of GPX4, ACSL4 and A20 proteins in HUVECs. (L) The exosomes extracted after transfection can affect the mitochondrial membrane potential of HUVECs (*p < 0.05; **p < 0.01; ***p < 0.001; ****p < 0.0001).

We collected and validated the exosomes of two types of gastric cancer cells HGC-27, and MKN-45 by ultracentrifugation. We found that all of them could express the marker proteins of exosomes (Fig. 5A) and appeared as vesicles with a diameter of about 100-150 nm under electron microscope (Fig. 5B). Through NTA instrument detection, we found that the diameters of collected exosomes were mostly enriched around 146 nm (Fig. 5C). In order to further determine the enrichment location of miR-214-3p, we detected the content of miR-214-3p in exosomes and DMEM culture medium from which exosomes were removed by PCR, and proved that it mainly exists in exosomes (Fig. 5D). Next, we transiently transfected miR-214-3p mimics, inhibitors and their controls into gastric cancer cells and extracted exosomes, respectively. These exosomes were co-cultured with HUVECs for 48 h, and total RNA and protein were extracted. We verified by PCR that co-culture could affect miR-214-3p expression in HUVECs (Figs. 5E–5G). And we verified by PKH26 staining experiment that the stained exosomes could enter HUVEC cells after 8 h of co-culture (Fig. 5H), confirming that it indeed changed the intracellular miR-214-3p and its miR-214-3p due to the uptake of exosomes. Expression of downstream A20/ACSL4 protein. To further validate the relationship between miR-214-3p and ferroptosis, we performed a series of experiments to confirm that up-regulated miR-214-3p could increase intracellular GSH (Fig. 5I), reduce accumulated lipid ROS (Fig. 5J). We then verified that the altered miR-214-3p could further regulate the expression of A20/ACSL4 by western blotting (Fig. 5K). Also, the up regulation of miR-214-3p can rise the intracellular mitochondrial membrane potential (Fig. 5L). We have verified the mutual regulation mechanism of miR-214-3p/A20/ACSL4 axis and its relationship with ferroptosis in HUVEC cells.

### Co-action of miR-214-3p inhibitors and Apatinib

**Figure 6:**
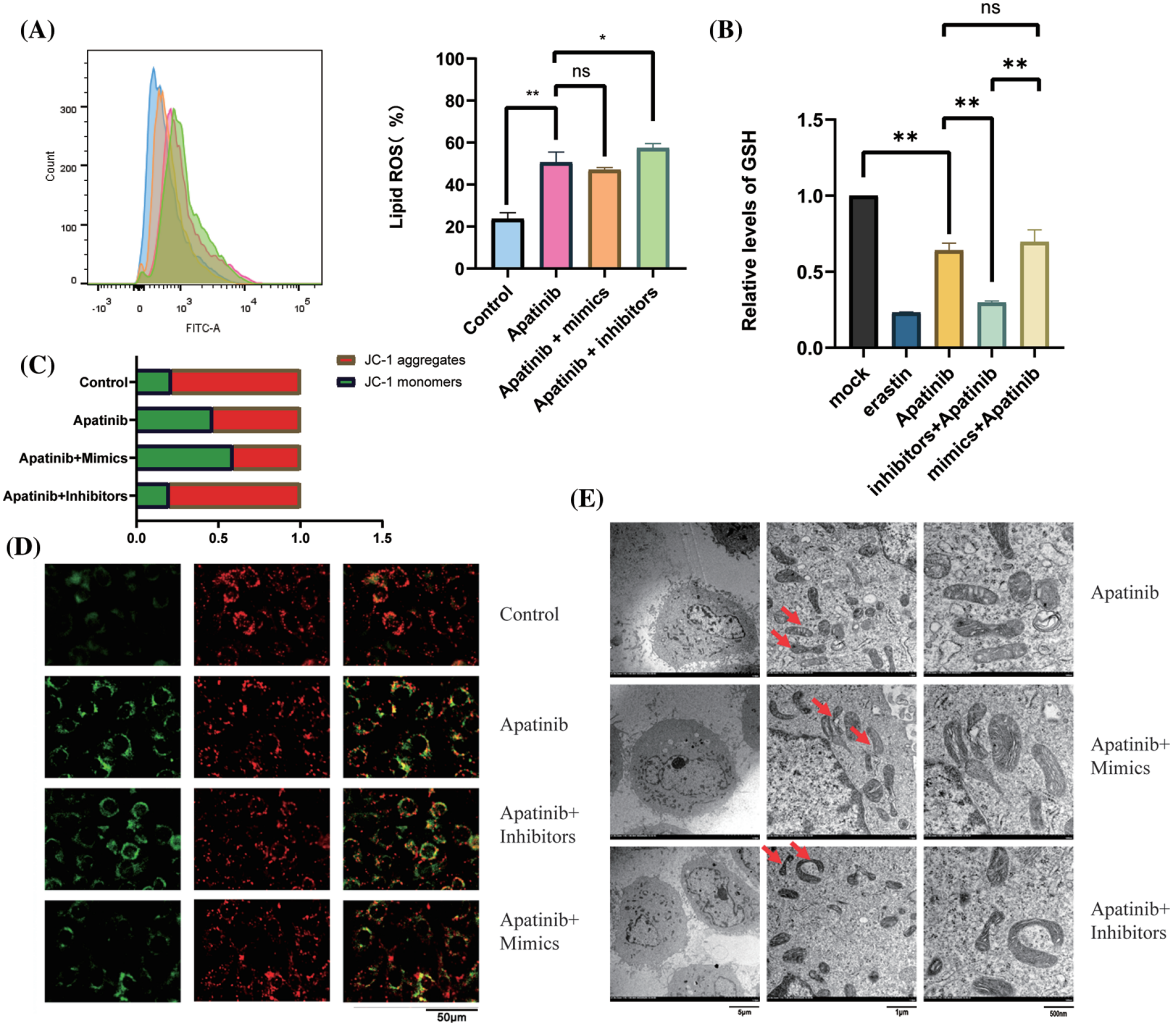
Co-action of miR-214-3p inhibitors and Apatinib. (A) Apatinib synergizes with miR-214-3p inhibitors to increase lipid ROS accumulation within HUVECs. (B) Apatinib synergized with miR-214-3p inhibitors to reduce GSH content in HUVECs. (C, D) Apatinib synergistically reduces HUVEC mitochondrial membrane potential with miR-214-3p inhibitors. (E) Mitochondrial shrinkage caused by Apatinib was observed under electron transmission microscopy with the addition of miR-214-3p inhibitors (*p < 0.05; **p < 0.01; ***p < 0.001; ****p < 0.0001).

Next, we wondered whether miR-214-3p co-acted with Apatinib. We added 150 μM Apatinib to HUVEC cells transfected with miR-214-3p mimics and inhibitors and observed their co-action after 24 h. We found that miR-214-3p inhibitors synergized with Apatinib to increase intracellular lipid ROS accumulation (Fig. 6A), decrease intracellular GSH (Fig. 6B) and decrease intracellular mitochondrial membrane potential (Figs. 6C and 6D). The addition of miR-214-3p inhibitors aggravated mitochondrial shrinkage under electron transmission microscopy (Fig. 6E). Intracellular transfection of miR-214-3p mimics could not reverse Apatinib-induced ferroptosis in HUVEC cells. These results demonstrate that miR-214-3p inhibitors can synergize the effect of Apatinib to exacerbate ferroptosis in HUVECs *in vitro*, thereby sensitizing the antiangiogenic effect of Apatinib.

### Validation of the anti-angiogenic effect of miR-214-3p inhibitors sensitizing Apatinib in vivo

**Figure 7:**
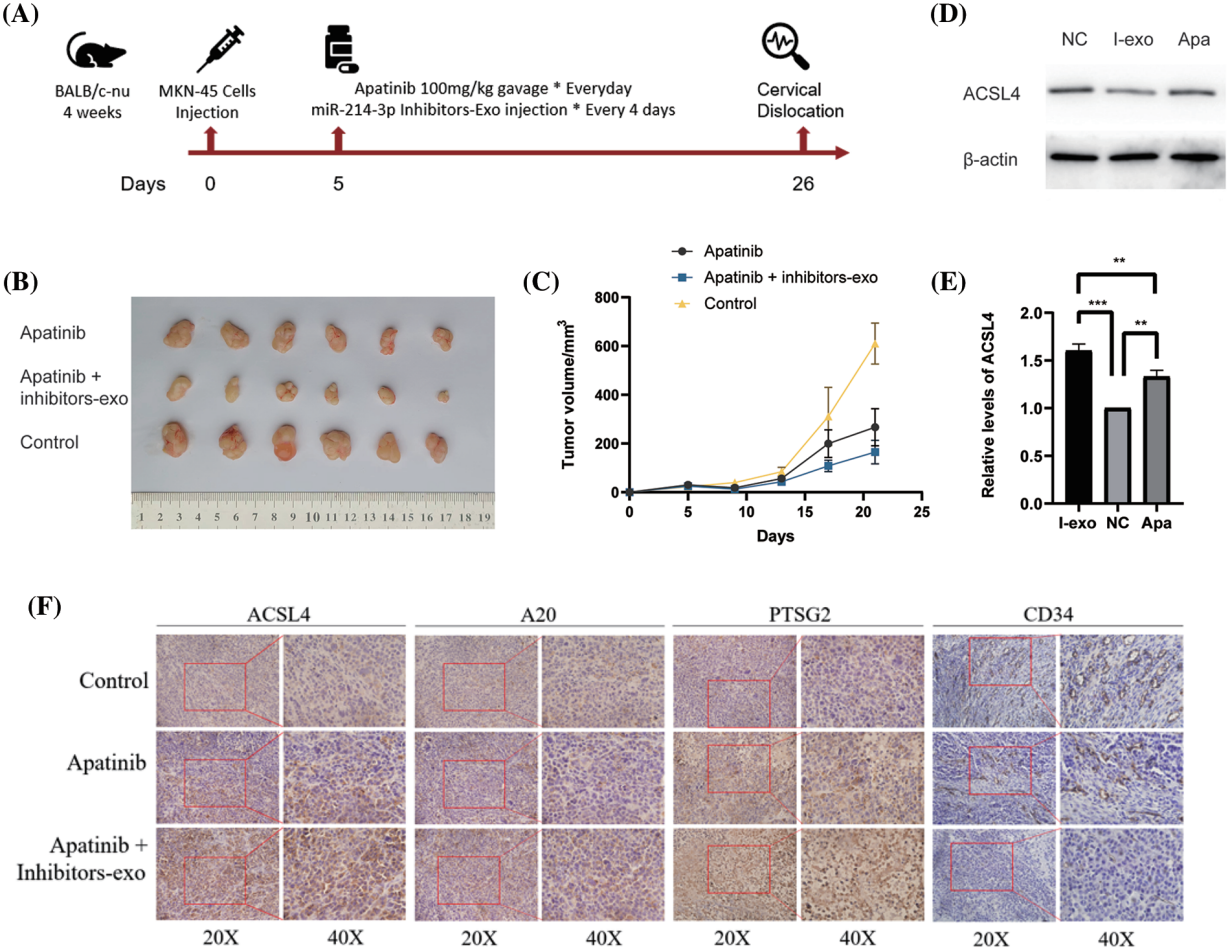
Validation of the anti-angiogenic effect of miR-214-3p inhibitors sensitizing Apatinib *in vivo*. (A) *In vivo* experimental procedure. (B) Tumors of three groups of nude mice. (C) Changes in tumor volume of three groups of nude mice. (D, E) ACSL4 content in tumor-extracted proteins. (F) The contents of ACSL4, A20, PTSG2 and CD34 in tumor sections of the three groups (*p < 0.05; **p < 0.01; ***p < 0.001; ****p < 0.0001).

Next, we used the MKN-45 gastric cancer cell line to perform a xenograft tumor model in the right groin of 4-week-old female nude mice. First, we massively expanded the MKN45 cell line, and then, we used PBS to re-suspend the cells and match them to the appropriate cell concentration. We drew cell suspension with an empty needle of 1ml and injected it into the skin of the mouse’s lower groin to form a pico. After 5 days of observation, the tumor was locally formed. We started to give the mice in the drug group a daily gavage of Apatinib five days after tumor formation, and injected exosomes in PBS containing miR-214-3p inhibitors into the tumors of the Apatinib + miR-214-3p inhibitors group every four days. Further analysis was performed after 21 days of dosing (Fig. 7A). We found that the tumor size of the Apatinib + miR-214-3p inhibitors group was significantly reduced (Figs. 7B and 7C), indicating that it can indeed synergize with Apatinib to inhibit tumor growth. We extracted the entire protein of the tumor and verified the ACSL4 expression of the tumor by western blotting, which was found to be consistent with *in vitro* (Figs. 7D and 7E). Next, we verified the expression of A20/ACSL4 in the tumor by immunohistochemistry, the expression of the ferroptosis marker PTGS2, and the obvious effect of Apatinib + miR-214-3p inhibitors on tumor angiogenesis by CD34. inhibition (Fig. 7F). We have confirmed the antitumor effect of miR-214-3p inhibitors synergistically with Apatinib from *in vivo* experiments.

## Discussion

Gastric cancer remains one of the major contributors to cancer deaths worldwide [39]. Many treatment strategies have been developed, but most patients are asymptomatic in the early stage. Therefore, gastric cancer has a poor prognosis with a 5-year survival rate of <10% [39]. Studies performed to date have indicated its significant role in a few human diseases, including its increased efficiency in neurodegenerative diseases, ischemic reperfusion injury, atherosclerosis, and cancer [40]. On the other hand, cell proliferation requires ferroptosis, which may indicate a physiological role in the process [41]. Acquired drug resistance is associated with a poor prognosis in patients with advanced tumors [42]. Accumulating evidence supports ferroptosis as a potential target for chemotherapy resistance. Pharmacological induction of ferroptosis could reverse drug resistance in tumors [43]. Ferroptosis is recognized as a promising strategy for overcoming resistance to chemotherapy, targeted therapy, immunotherapy, and radiation therapy in cancer.

Lipid peroxidation is a symbolic process of ferroptosis, whose main targets are polyunsaturated fatty acids (PUFAs) produced by lipid synthesis [44]. Reactive oxygen species (ROS) are required for most normal biological processes [45], but are also related to ferroptosis when it's excessively accumulated [46]. As the main facilitator of ferroptosis, the synthesis of cell membrane by PUFAs and the iron-containing enzyme lipoxygenase relies most on the function of ACSL4, which prefers long polyunsaturated fatty acids such as AA and AdA [8]. Suppression of AA or AdA esterification into PE by ACSL4 acts as a specific anti-ferroptotic rescue pathway [47]. ACSL4 esterifies CoA to free fatty acids in an ATP-dependent way, activating them for oxidation or lipid biosynthesis [8]. ACSL4 is upregulated in several cancers including hepatocellular carcinoma, colorectal cancer, prostate cancer, and breast cancer [48–50]. However, the expression of ACSL4 is frequently down-regulated in gastric cancer, increasing cell growth and cell migration, and further studies are needed to explain these differences [51]. It has been reported that ACSL4 plays a tumor-suppressive role and could be a potential therapeutic target in GC [51].

A20, also named TNF-α-induced protein 3 (TNFAIP3), is a potent regulator of ubiquitin (Ub) dependent signals, which is a ubiquitin editing enzyme with both deubiquitinating enzyme (DUB) activity and E3 ubiquitin ligase activity [52–56]. Modifying targets proteins for degradation with K48-linked polyubiquitin chains and stimulates the recruitment of signaling proteins with K63-linked polyubiquitin chains [57–59]. Therefore, A20 modifies ubiquitylated protein substrates in multiple ways. ZF4, A20’s fourth zinc finger, binds K63-linked polyubiquitin chains and acts as E3 ligase [58].

**Figure 8:**
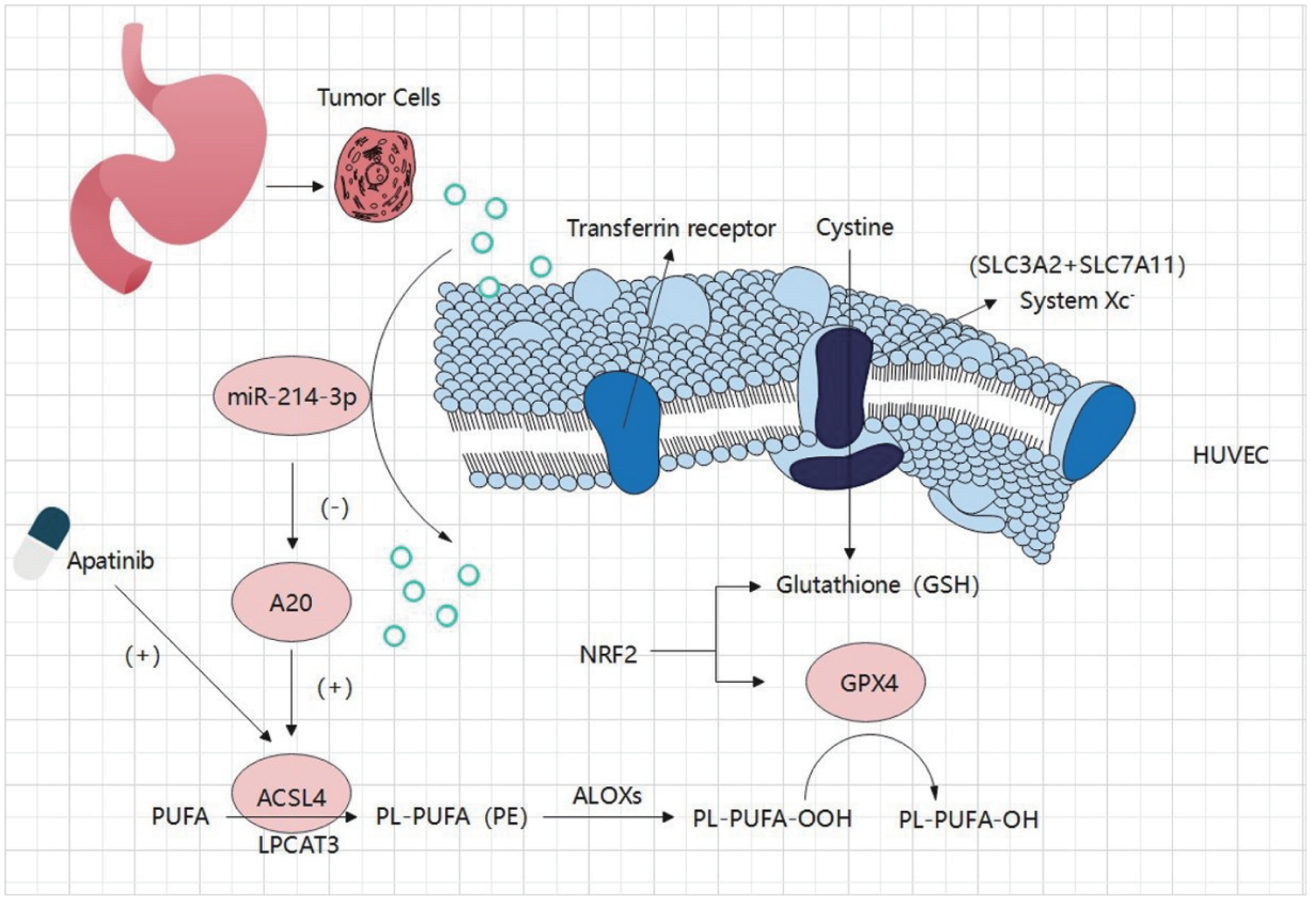
Gastric cancer secreted miR-214-3p inhibits the anti-angiogenesis effect of Apatinib by suppressing ferroptosis in vascular endothelial cells. The exosomes secreted by gastric cancer cells carry a large amount of miR-214-3p, which enters vascular endothelial cells and releases its contents. The amount of miR-214-3p in vascular endothelial cells increases, regulating the occurrence of ferroptosis in vascular endothelial cells through the miR-214-3p/A20/ACSL4 axis. At the same time, Apatinib can directly act on ACSL4 in vascular endothelial cells. As a result, miR-214-3p resulted in the failure of Apatinib.

In our study, we first identified Apatinib as a ferroptosis inducer in vascular endothelial cells and a standard drug for third-line treatment in advanced gastric cancer. This makes the path for, as the mechanism of action of anti-angiogenesis drugs increased significantly meaningful a content. Apatinib affects the peroxidation of fatty acids in cells by causing changes in ACSL4 levels in cells, which alters the quantitative accumulation of lipid peroxides in cells and affects ferroptosis in cells. In this study, we investigated at least two mechanisms that affect ferroptosis in vascular endothelial cells via an important target, ACSL4 (Fig. 8). One of them is that miR-214-3p, which is highly expressed in gastric cancer cells, is carried in the exosomes released by gastric cancer cells into the tumor microenvironment, which is ingested into vascular endothelial cells in the tumor microenvironment, resulting in a large number of miR-214-3p released from exosomes, resulting in a synchronous increase in the level of miR-214-3p in vascular endothelial cells. Upregulation of miR-214-3p significantly inhibited the expression of ubiquitin editing enzyme A20 in vascular endothelial cells. The up-regulation of miR-214-3p has a variety of effects in cells, and in this case, it will affect a variety of physiological processes in cells at the same time, including the common PTEN-related tumor suppressor mechanism, A20/NF-κB related inflammatory mechanism, and so on. We found that A20 functions as a deubiquitinating enzyme for ACSL4, which was reflected in the fact that miR-214-3p inhibited the expression of A20 to reduce its protective effect on the ubiquitination and degradation of ACSL4, resulting in the degradation of ACSL4 in cells. In this way, the reduction of ACSL4 caused by miR-214-3p formed a hedge against the effect of Apatinib, making Apatinib unable to exert its full effect to inhibit angiogenesis, which was manifested as drug resistance.

The decrease of miR-214-3p itself can inhibit the growth and proliferation of gastric cancer cells. Based on the above findings, our study also found that inhibiting the expression of miR-214-3p can reduce the occurrence of Apatinib resistance. Therefore, we inspired the subsequent research direction, and the appropriate uptake of miR-214-3p inhibitors in humans will be a powerful adjuvant therapy for patients with gastric cancer. The role of A20 as an epigenetic regulator of proteasome-dependent degradation in ferroptosis was also explored through the intermediate mechanism study, which provided an example for future research.

In this study, we explored the function of Apatinib in the induction of ferroptosis in vascular endothelial cells by regulating ACSL4. The miRNA expression profile of advanced gastric cancer was studied, and miR-214-3p was identified as one of the key miRNAs regulating ferroptosis in vascular endothelial cells. We questioned its underlying mechanism-the miR-214-3p/A20/ACSL4 axis. Subsequently, we verified the synergistic therapeutic effect of knockdown of miR-214-3p combined with Apatinib in gastric cancer *in vitro*. The results presented here illustrate a new molecular mechanism to understand how Apatinib sensitizes vascular endothelial cells to ferroptosis can reverse the drug resistance and provide a potentially feasible combination therapy for gastric cancer treatment.

## Supplementary Materials

Supplementary figure 1 Increased endocytosis and exocytosis, accumulation of giant mitochondria and lipid droplets in Apatinib-treated HUVEC under electron microscopy.

Supplementary figure 2 Immunohistochemical expression of A20 in cancer tissues and in paracancerous tissues.

Supplementary figure 3 Complete miRNA differential expression profiles obtained from GEO database analysis.

## Data Availability

The datasets used or analysed during the current study are available from the corresponding author on reasonable request.

## Supplementary Materials

The supplementary material is available online at https://doi.org/10.32604/or.2023.046676.
