# Supplementary figures and images for: Gastric cancer secreted miR-214-3p inhibits the anti-angiogenesis effect of apatinib by suppressing ferroptosis in vascular endothelial cells
Source: Oncol Res. 2024 Feb 6;32(3):489–502. doi: 10.32604/or.2023.046676 (PMC10874472; doi:10.32604/or.2023.046676)

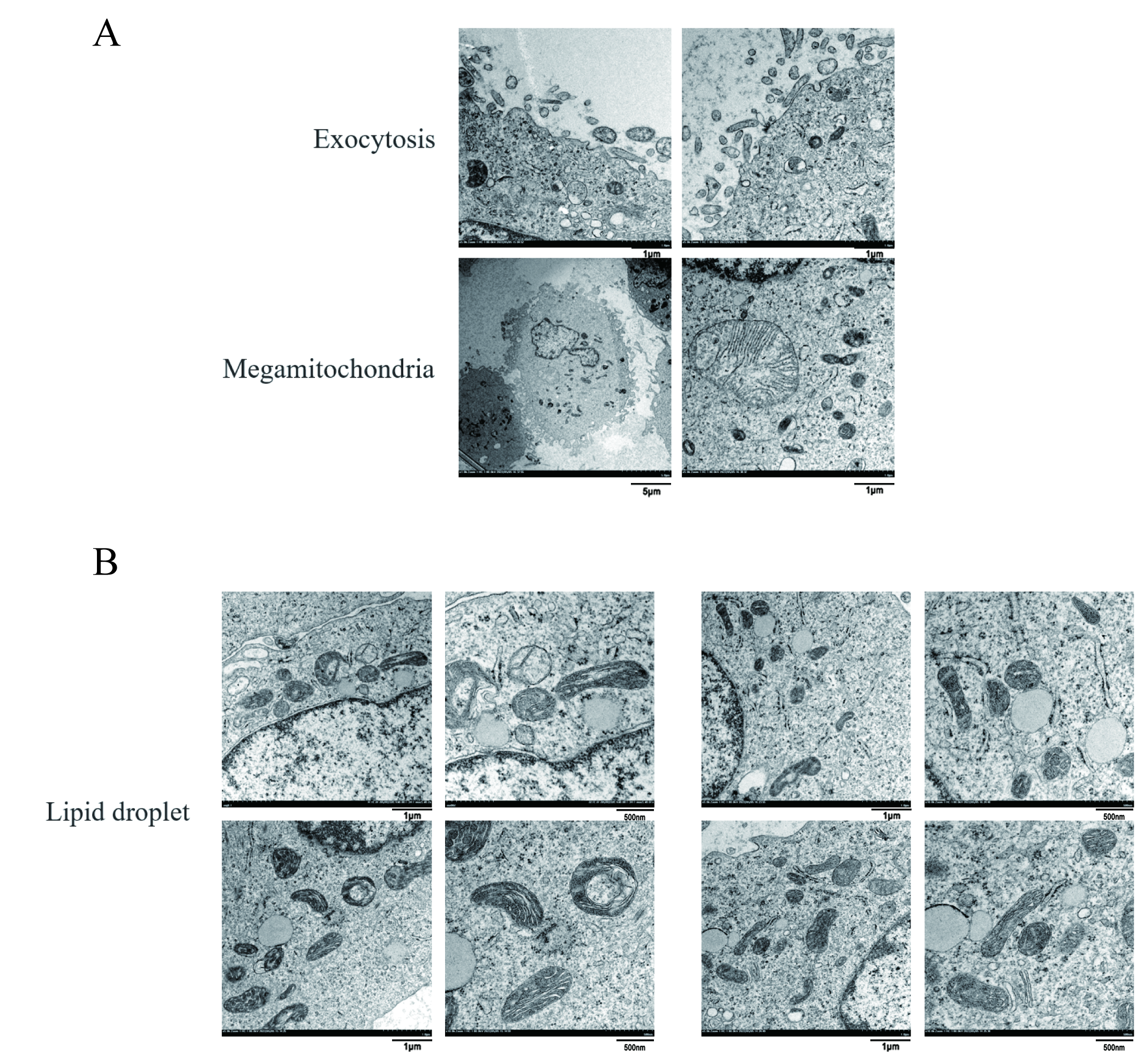

Supplement: Supplementary figure 1 [file OncolRes-32-46676-s001.tif]

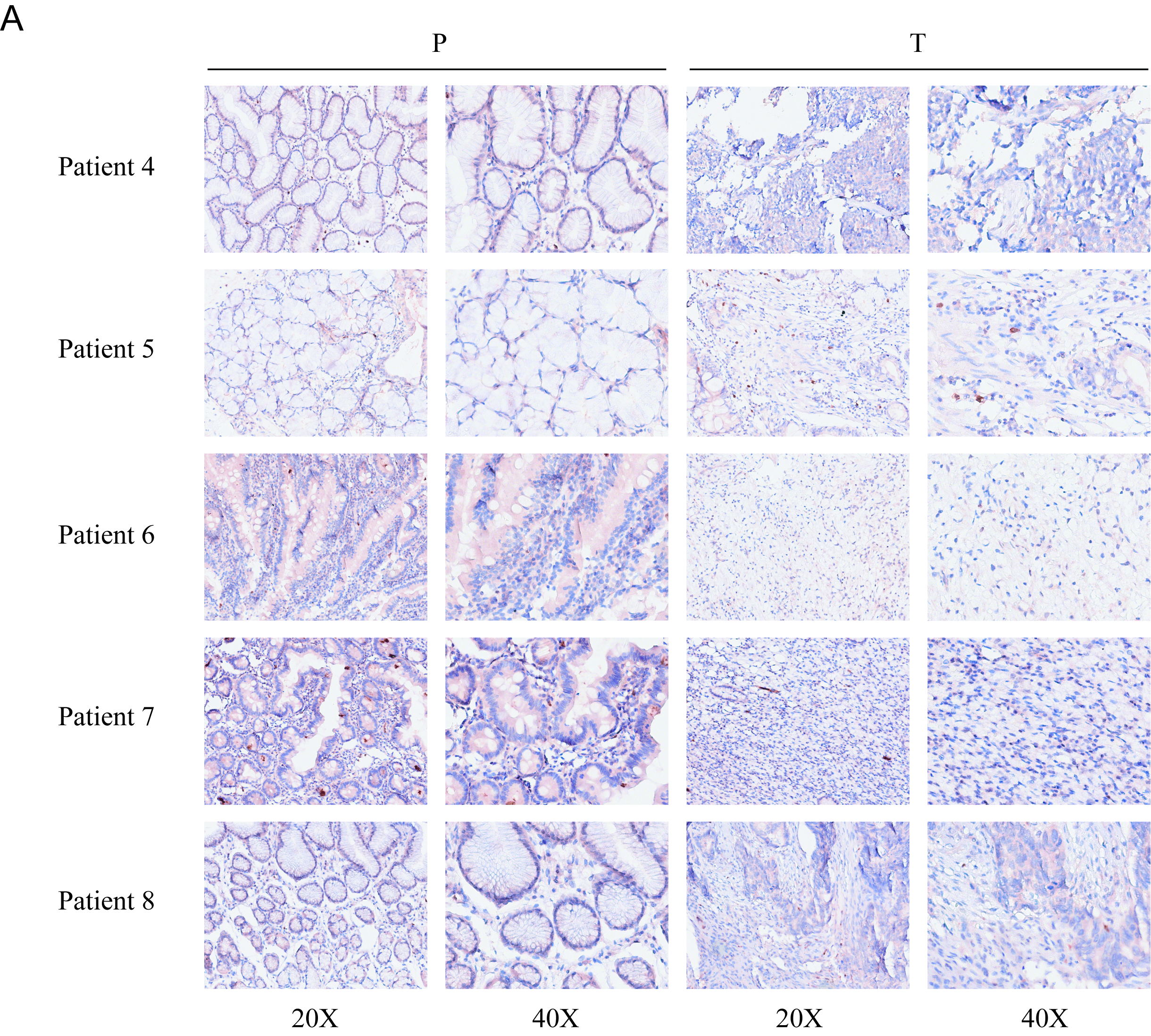

Supplement: Supplementary figure 2 [file OncolRes-32-46676-s002.tif]

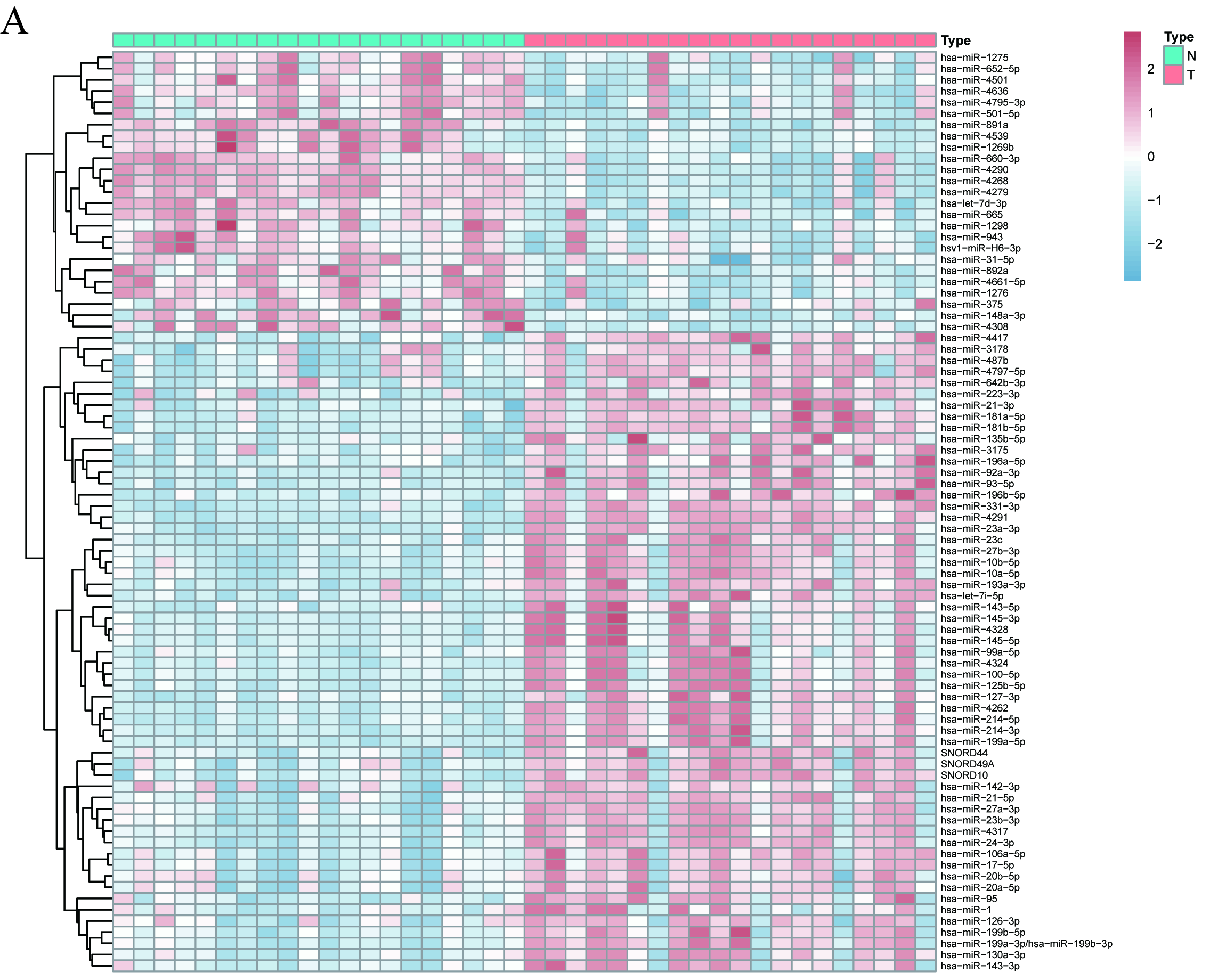

Supplement: Supplementary figure 3 [file OncolRes-32-46676-s003.tif]
